# Transcranial direct current stimulation–induced changes in motor cortical connectivity are associated with motor gains following ischemic stroke

**DOI:** 10.1038/s41598-024-66464-5

**Published:** 2024-07-08

**Authors:** Chunfang Wang, Xiangli Yang, Dan Guo, Weiguang Huo, Ningbo Yu, Ying Zhang

**Affiliations:** 1 Rehabilitation Medical Department, Tianjin Union Medical Centre, Tianjin, China; 2 Tianjin Institute of Rehabilitation, Tianjin, China; 3 Tianjin Key Specialty of Spinal Rehabilitation with Integrated Traditional Chinese and Western Medicine, Tianjin, China; 4 Otolaryngological Department, Tianjin Union Medical Centre, Tianjin, China; 5 College of Artificial Intelligence, Nankai University, Tianjin, China

**Keywords:** Stroke, Motor gain, tDCS, EEG, Connectivity, Coherence, Neuronal physiology, Neurology, Predictive markers, Geriatrics

## Abstract

Understanding the response of the injured brain to different transcranial direct current stimulation (tDCS) montages may help explain the variable tDCS treatment results on poststroke motor gains. Cortical connectivity has been found to reflect poststroke motor gains and cortical plasticity, but the changes in connectivity following tDCS remain unknown. We aimed to investigate the relationship between tDCS-induced changes in cortical connectivity and poststroke motor gains. In this study, participants were assigned to receive four tDCS montages (anodal, cathodal, bilateral, and sham) over the primary motor cortex (M1) according to a single-blind, randomized, crossover design. Electroencephalography (EEG) and Jebsen-Taylor hand function test (JTT) were performed before and after the intervention. Motor cortical connectivity was measured using beta-band coherence with the ipsilesional and contralesional M1 as seed regions. Motor gain was evaluated based on the JTT completion time. We examined the relationship between baseline connectivity and clinical characteristics and that between changes in connectivity and motor gains after different tDCS montages. Baseline functional connectivity, motor impairment, and poststroke duration were correlated. High ipsilesional M1**–**frontal–temporal connectivity was correlated with a good baseline motor status, and increased connectivity was accompanied by good functional improvement following anodal tDCS treatment. Low contralesional M1**–**frontal-central connectivity was correlated with a good baseline motor status, and decreased connectivity was accompanied by good functional improvement following cathodal tDCS treatment. In conclusion, EEG-based motor cortical connectivity was correlated with stroke characteristics, including motor impairment and poststroke duration, and motor gains induced by anodal and cathodal tDCS.

## Introduction

Motor impairment is a leading cause of disability following stroke. The performance of daily activities such as eating, dressing and self-care, requires the use of the upper limbs, and upper limb dysfunction severely affects the ability to perform activities of daily living. Despite receiving rehabilitation, 40–50% of patients with stroke have persistent upper limb functional deficits^1^, underlining the need to develop more effective neurorehabilitation treatments for motor relearning.

According to the interhemispheric competition model of stroke recovery, impairment can be reduced and functional recovery can be promoted by applying non-invasive brain stimulation (NIBS) to increase ipsilesional corticospinal excitability or decrease contralesional corticospinal excitability^2^. Thus, the potential role of NIBS in brain and motor recovery poststroke has been of particular interest to researchers in the field of stroke rehabilitation. Due to the modulating effects of transcranial direct current stimulation (tDCS) on cortical excitability, especially when applied to the primary motor cortex (M1), tDCS has been widely used to aid motor relearning following stroke^3^. To achieve the normal interhemispheric inhibition, tDCS therapy aims to increase ipsilesional M1 excitability, decrease contralesional M1 excitability, or do both simultaneously (via bilateral tDCS), and it has been found to augment motor gains during rehabilitation following stroke^4–6^.

However, clinical trials of tDCS for stroke recovery have shown high interpatient variability, with some patients showing no benefit^7–9^. tDCS is not considered a one-size-fits-all intervention^10,11^. Different tDCS montages may benefit different stoke patients, as different tDCS montages may induce diverse effects on brain cortical networks depending on the position and polarity of the electrodes^12^. Heterogeneous clinical characteristics of the study participants may also explain the variable results of the effect of tDCS. Factors like age, poststroke duration, and impairment severity may shape the response to tDCS^11,13,14^. Understanding the brain network response to different tDCS montages and its relationship with clinical characteristics may help us explain the variability in motor gains in response to different tDCS montages in patients with stroke.

Quantitative electroencephalography (EEG) is an established method for assessing the functional state of the brain^15^. EEG-based measures of connectivity have been reported to predict motor gains in both healthy individuals and patients with stroke^16,17^. EEG frequency analysis can provide information on the oscillations in each frequency band. Beta-frequency oscillations are associated with motor function^18,19^. Assessment of high-frequency beta oscillations showed that strong connectivity of the stimulated sensorimotor network was associated with a great increase corticospinal excitability following a facilitatory tDCS montage in healthy individuals^20^. In addition, beta coherence, a measure of connectivity, was found to be a robust biomarker of cortical function and plasticity after stroke^17^. Consequently, we hypothesized that cortical connectivity, measured in terms of beta coherence, would be correlated with the clinical characteristics of patients with stroke and that these patients would show different responses in terms of motor gains to different tDCS montages. Understanding how tDCS montages and clinical characteristics affect the reorganization of brain connectivity and the interaction between motor gains and changes in connectivity following tDCS can aid individualized tDCS montage selection for promoting motor gains after stroke.

## Results

### Safety and completion of the tDCS sessions

The four tDCS montages was well tolerated by all the participants; no participated reported any side effects in the post-intervention questionnaire. One participant was unable to participate her last experiment session (atDCS session) due to personal reasons. This participant’s data was not included when analyzing the effect of atDCS, but was included when analyzing the effect of the other three tDCS montages.

### Participants

This study included 28 participants (nine women and nineteen men), aged 39–78 (58.5 ± 9.91) years, who were in the chronic phase of stroke recovery (mean poststroke duration: 13.6 ± 7.38 months, range 5–26 months). All the participants had mild to moderate upper limb motor impairment (mean UEFM score: 50.6 ± 8.56, range 36–64; mean MBI score: 91.8 ± 10.11, range 60–100). Table 1 shows the demographic and clinical characteristics of the participants.

**Table 1:** Participant characteristics in this study.

Subject	Gender	Age (/y)	Hand	Hemisphere	Cite	Duration (/m)	UEFM	MBI	MAL:AOU	MAL:QOM
1	Male	63	Right	Right	BG	21	55	90	38	58
2	Female	60	Right	Right	BS	9	57	80	116	116
3	Male	39	Right	Left	BS	8	59	100	57	101
4	Male	63	Right	Left	BS	13	41	70	2	4
5	Male	64	Right	Left	BG	20	57	100	74	114
6	Male	56	Right	Right	BG	21	50	90	7	7
7	Male	47	Right	Left	BG	11	57	90	74	96
8	Female	63	Left	Left	BG	6	64	100	113	115
9	Male	61	Left	Right	BG	21	61	100	150	145
10	Male	61	Right	Right	BG	24	44	100	23	23
11	Male	56	Right	Left	BG	26	60	100	90	90
12	Male	59	Right	Right	BG	5	63	100	120	121
13	Male	44	Left	Left	BG	5	46	85	39	32
14	Male	40	Right	Right	BG	5	63	100	112	133
15	Female	68	Right	Left	BG	4	53	85	29	37
16	Male	46	Right	Left	BG	23	44	90	68	78
17	Female	72	Right	Left	BS	8	36	95	71	63
18	Male	55	Right	Left	BS	8	60	100	110	116
19	Female	62	Right	Left	BS	12	44	80	76	107
20	Male	45	Right	Left	BG	11	44	95	56	92
21	Female	62	Right	Left	BG	5	46	60	124	129
22	Female	69	Right	Right	BG	13	36	95	37	97
23	Female	65	Right	Right	BG	10	43	90	19	86
24	Male	65	Right	Right	BG	43	38	100	32	95
25	Male	53	Right	Right	BG	56	45	100	30	76
26	Female	71	Left	Right	BG	15	49	90	83	104
27	Male	78	Right	Right	BG	7	50	85	94	98
28	Male	51	Right	Right	BS	17	52	100	25	117

Age (year, y); Hand: Dominant hand; Hemisphere: Hemisphere affected by stroke; Cite = Cite of lesion; BG: Basal ganglia; BS: Brain stem; Duration = Poststroke duration(/month, m); *UEFM* Upper Extremity Fugl-Meyer scores, *MBI* Modified Barthel Index, *MAL* Motor activity log, *AOU* Amount of use, *QOM* Quality of movement.

### Upper limb motor function

**Figure 1:**
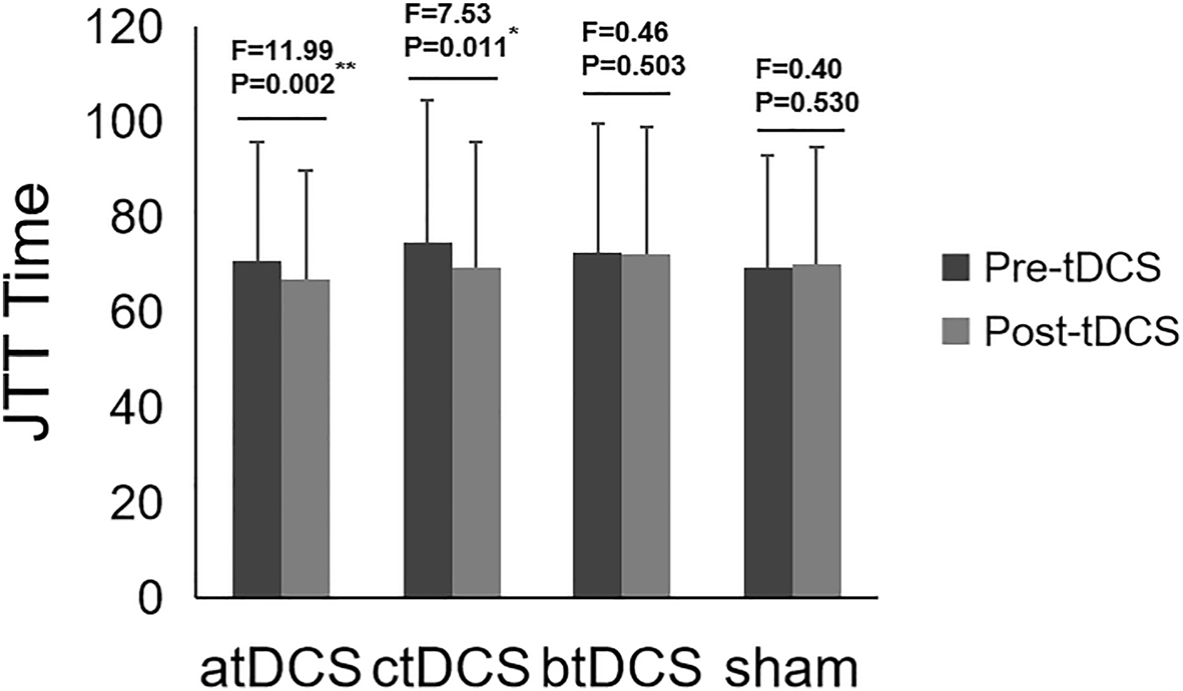
JTT times of the four tDCS montage groups. *JTT* Jebsen–Taylor Hand Function Test, *atDCS* anodal tDCS, *ctDCS* cathodal tDCS, *btDCS* bilateral tDCS, *sham* sham tDCS.

Table 2 shows the Within-Subjects Effects of JTT time using Two-way repeated ANOVA. Supplemental Fig. 1 shows the estimated marginal means of JTT time of the four tDCS montages. The testing result showed a significant interaction between tDCS montages and stimulation treatment results. So, a separate effect analysis was performed after that. The results showed that JTT performance significantly improved after atDCS and ctDCS, but not after btDCS and sham stimulation. The separate effect analysis result of JTT times of the four tDCS montage groups are shown in Fig. 1. In order to demonstrate that participants get stable JTT performance prior to testing, we showed the 10 times practise result in Supplemental Fig. 2 and the statistical analyses result in supplemental Table 1. The repeated ANOVA result of the last three times practise showed no significant difference, indicating a relatively stable level before testing.

**Table 2:** Within-subjects effects of JTT time using two-way repeated ANOVA.

Sources	Type III Sum of Squares	df	F	Sig
Montage	628.237	1.391	0.710	0.450
Treatment	294.521	1.000	9.213	0.005**
Montage*Treatment	290.751	2.122	5.810	0.004**

Montage: Four tDCS montages (anodal transcranial direct current stimulation, atDCS; cathodal transcranial direct current stimulation, ctDCS; bilateral transcranial direct current stimulation, btDCS and sham tDCS); Treatment: Pre-stimulation and post-stimulation treatments.

** *p* < 0.01.

### Motor status and poststroke duration are associated with baseline connectivity

**Figure 2:**
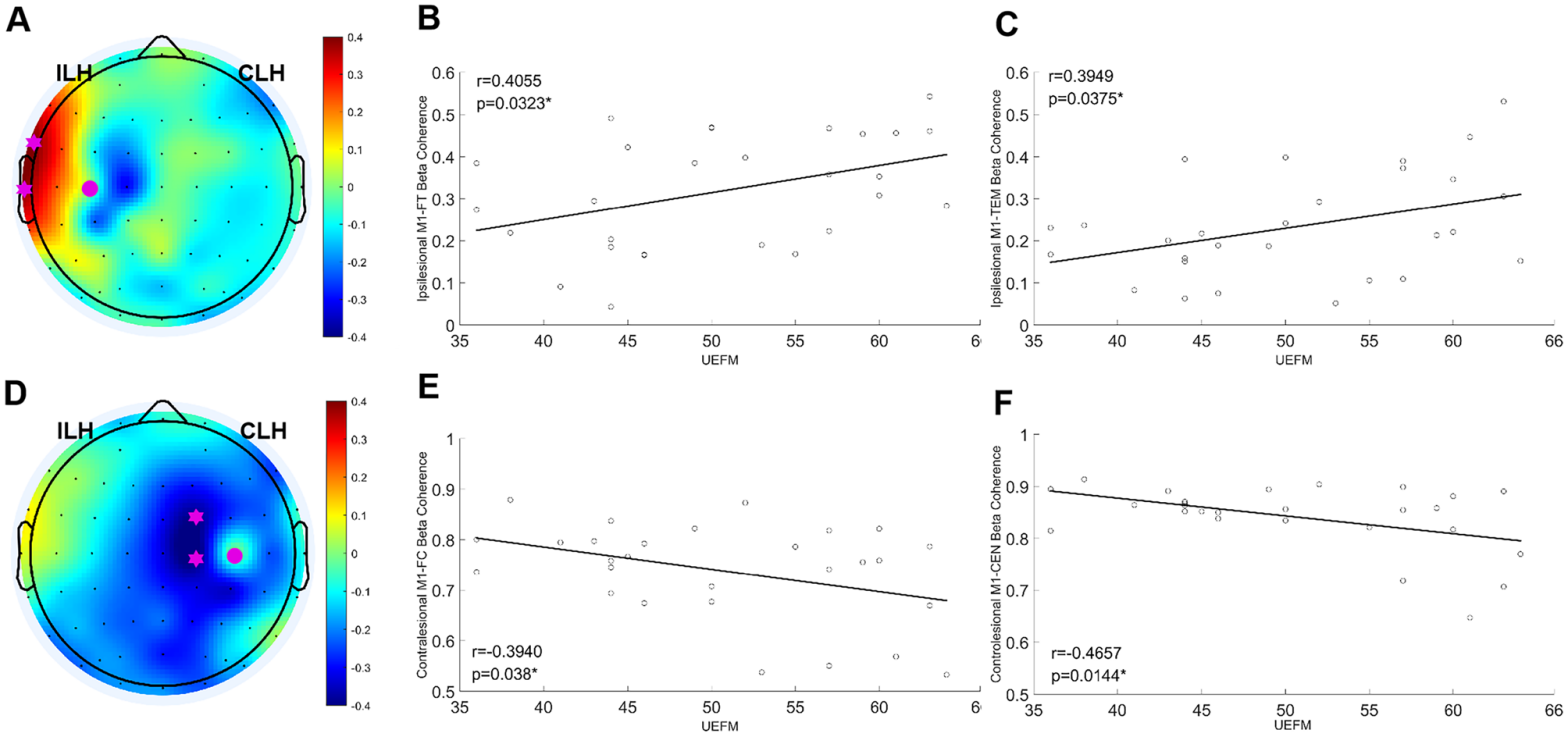
Correlation between cortical connectivity and motor status at baseline. (**A**) Topographic map of correlation coefficients between beta coherence at the ipsilesional M1 seed and UEFM score. Pink dots represent the ipsilesional M1 seed and pink stars represents areas showing statistically significant correlation between beta coherence and UEFM scores (*p* < 0.05). (**B**) Correlation of beta connectivity between the ipsilesional M1 and the frontal–temporal region with motor impairment (UEFM score). (**C**) Correlation of beta connectivity between the ipsilesional M1 and the temporal region with motor impairment (UEFM score). (**D**) Topographic map of correlation coefficients between beta coherence at the contralesional M1 seed and UEFM score. (**E**) Correlation of beta connectivity between the contralesional M1 and the frontal-central region with motor impairment (UEFM score). (**F**) Correlation of beta connectivity between the contralesional M1 and the central region with motor impairment (UEFM score). M1: primary motor cortex, *UEFM* upper extremity fugl-meyer assessment, *ILH* ipsilesional hemisphere, *CLH* contralesional hemisphere, *FT* frontal–temporal, *TEM* temporal, *FC* frontal-central, *CEN* central.

Beta coherence of the ipsilesional frontal–temporal (r = 0.4055, *p* = 0.0323) and temporal (r = 0.3949, *p* = 0.0375) cortices with the ipsilesional M1 was positively correlated with UEFM score. Beta coherence of the contralesional frontal-central (r = − 0.3940, *p* = 0.038) and central (r = − 0.4657, *p* = 0.0144) cortices with the contralesional M1 was negatively correlated with UEFM score (Fig. 2).

**Figure 3:**
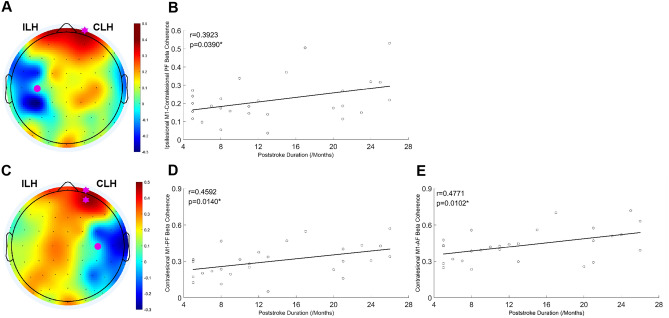
Correlation between cortical connectivity and poststroke duration at baseline. (**A**) Topographic map of the correlation coefficients between beta coherence at ipsilesional M1 seed and upper poststroke duration. Pink dots represent the ipsilesional M1 seed and pink stars represent areas showing statistically significant correlation between beta coherence and poststroke duration (*P* < 0.05). (**B**) Correlation of beta connectivity between the ipsilesional M1 and the contralesional prefrontal region with poststroke duration. (**C**) Topographic map of the correlation coefficients between contralesional M1 seed and poststroke duration. (**D**) Correlation of beta connectivity between the contralesional M1 and prefrontal region with poststroke duration. (**E**) Correlation of beta connectivity between the contralesional M1 and frontal region with poststroke duration. *M1* primary motor cortex, *ILH* ipsilesional hemisphere, *CLH* contralesional hemisphere, *PF* prefrontal.

Beta coherence of the contralesional prefrontal cortex (r = 0.3923, *p* = 0.0390) with the ipsilesional M1 was positively correlated with poststroke duration, and beta coherence of the contralesional prefrontal (r = 0.4592, *p* = 0.0140) and frontal (r = 0.4771, *p* = 0.0102) cortices with the contralesiona M1 was positively correlated with poststroke duration (Fig. 3).

The baseline connectivity was not significantly correlated with the MBI score and age (*p* > 0.05).

### Changes in cortical connectivity induced by atDCS and ctDCS are associated with motor gains

**Figure 4:**
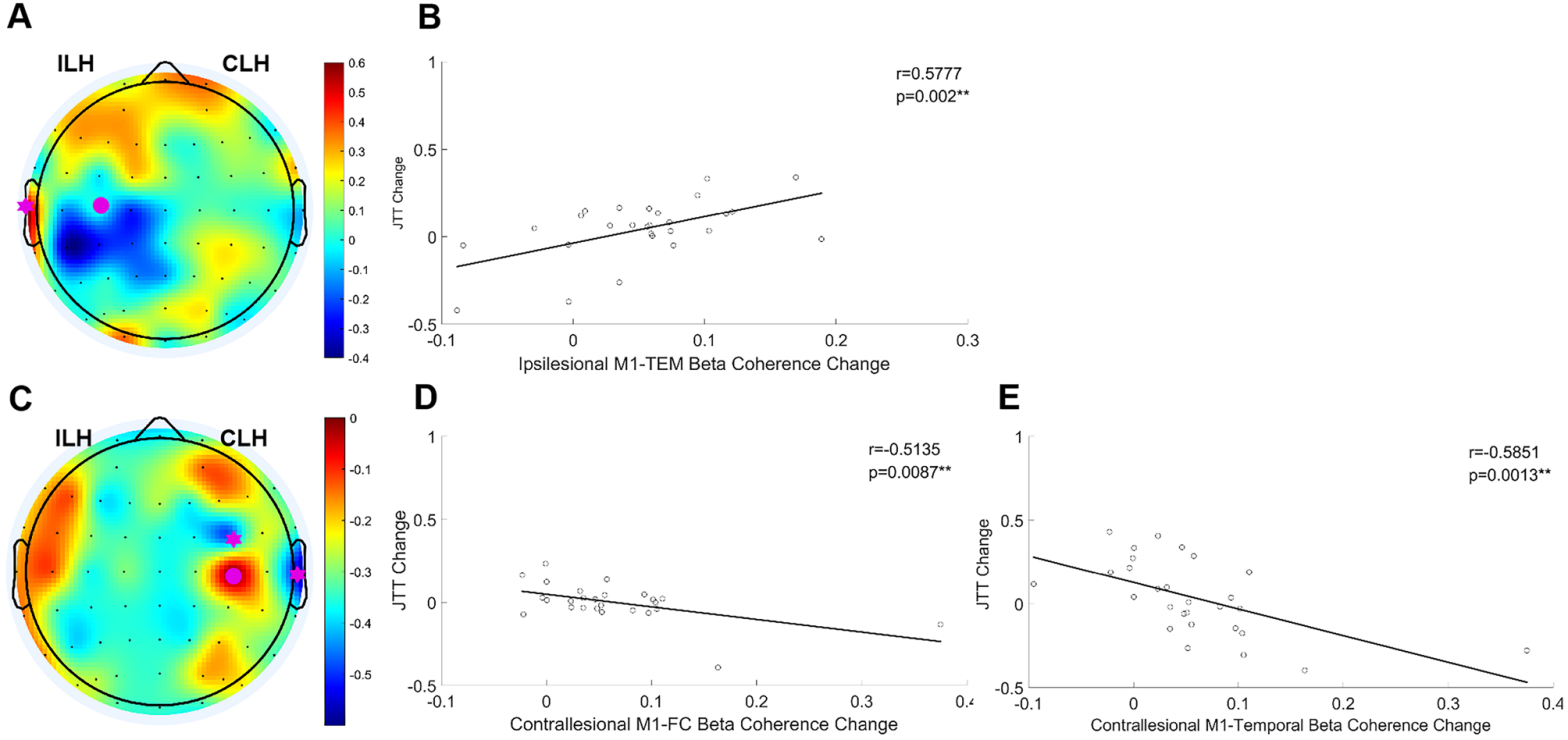
Correlation between changes in cortical connectivity following anodal and cathodal tDCS and motor gains. (**A**) Topographic map of the correlation coefficients between the change in beta connectivity with ipsilesional M1 seed after anodal tDCS and improvement in JTT time. Pink dots represent the ipsilesional M1 seed and pink stars represent areas showing statistically significant correlation between the change of beta coherence and JTT time (*P* < 0.05). (**B**) Correlation of change in connectivity between the ipsilesional M1 and the temporal region and the improvement in JTT time. (**C**) Topographic map of the correlation coefficients between the change in beta connectivity with contralesional M1 seed after cathodal tDCS and improvement in JTT time. (**D**) Correlation of change in connectivity between the contralesional M1 and frontal-central region with improvement in JTT time. (**E**) Correlation of change in connectivity between the contralesional M1 and temporal region with improvement in JTT time. *tDCS* transcranial direct current stimulation, *M1* primary motor cortex, *JTT* Jebsen–Taylor Hand Function Test, *ILH* ipsilesional hemisphere, *CLH* contralesional hemisphere, *TEM* temporal, *FC* frontal-central.

Improvement in JTT time after atDCS was correlated with the increase in ipsilesional M1-temporal connectivity (r = 0.5777, *p* = 0.002), and improvement in JTT time after ctDCS were correlated with the decrease in contralesional M1–frontal-central (r = 0.5135, *p* = 0.0087) and M1-temporal (r = − 0.5851, *p* = 0.0013) connectivity (Fig. 4). The change in connectivity after btDCS and sham did not correlate with the change in JTT time.

## Discussion

This study showed that high ipsilesional M1–frontal–temporal connectivity and low contralesional M1–frontal-central connectivity correlated with good baseline motor status. A previous study reported that beta coherence between the ipsilesional M1 and frontal-premotor cortical region was associated with motor impairment in patients with chronic stroke^17^, which is consistent with our findings. The participants recruited in this study were required to be able to complete the JTT task, therefore, the sample population was relatively mild in this study. According to the bimodal balance-recovery model^10^, patients with mild injury follow interhemispheric competition model, posits that, after the brain injury caused by stroke, balance between the two hemispheres is disrupted, resulting in a decrease in excitability in ipsilesional hemisphere and an increase in excitability in contralesional hemisphere. This might account for the positive correlation between ipsilesional M1-frontal–temporal connectivity and negative correlation between the contralesional M1–frontal-central connectivity and motor impairment. We also found a correlation between baseline cortical connectivity and poststroke duration. Therefore, we controlled for poststroke duration when we subsequently investigated the relationship between motor gains from tDCS and change in cortical connectivity.

The JTT shows good validity and reliability for the assessment of functional hand motor skills^21,22^, and it has been used to evaluate the effect of non-invasive brain stimulation on upper limb motor gains in patients with stroke^4,23^. Previous studies have demonstrated a strong correlation between the improvement in JTT time and functional gains during poststroke rehabilitation^24,25^. Therefore, the JTT is an effective measure of upper limb motor gains in the recovery process following stroke. Our findings demonstrated that atDCS and ctDCS induce significant modulations in the cortical connectivity, which is improves the motor gains in the paretic hand of patients with chronic stroke.

Studies have shown that tDCS could facilitate cortical plasticity elicited by motor training^26^ and induce motor function improvement^27^. However, a systematic review mentioned that the motor gains promoted by different tDCS montages have not been adequately researched^12,28^. Nevertheless, most previous studies demonstrated that atDCS over the ipsilesional M1 was effective for motor rehabilitation in patients with chronic stroke^29,30^, which is consistent with our findings. Studies have shown inconsistent results of ctDCS and btDCS for motor recovery^6,31,32^, whereas we observed motor gains following ctDCS but not btDCS. This might be explained by differences in motor tasks and the time sequence from tDCS to motor task performance^33,34^. To further understand this, the cortical connectivity response to different tDCS montages should be studied.

Increased connectivity between the ipsilesional premotor and M1 areas have been associated with good motor outcomes^35,36^, suggesting that good poststroke motor outcomes correlate with enhanced functional connectivity within the ipsilesional hemisphere. Another recent study found greater increase in MRI-based interhemispheric functional connectivity in greater improvement in UEFM in moderate to severe stroke subjects^37^, supporting the correlation between motor related cortical connectivity and rehabilitation outcomes. Additionally, beta coherence was found to be a powerful predictor of motor gains during rehabilitation^17^. Good poststroke motor recovery was associated with increased activation of the ipsilesional regions related to motor function^38^. Consistent with these reports, we found that beta coherence between the ipsilesional M1 and temporal region was positively correlated to motor gains, and beta coherence between the contralesional M1 and frontal-central and temporal regions was negatively correlated to motor gains.

According to the prevailing model of poststroke interhemispheric imbalance, atDCS could upregulate excitability of the ipsilesional M1 to promote neural plasticity, ctDCS could downregulate excitability of the contralesional M1 to achieve the same effect^12^, and btDCS could simultaneously upregulate ipsilesional M1 excitability and downregulate the contralesional M1 excitability. This study showed that atDCS promoted motor learning by enhancing the intra-hemispheric connectivity of the ipsilesional hemisphere and ctDCS promoted motor learning by reducing the intra-hemispheric connectivity of the contralesional hemisphere. Our findings suggest that atDCS and ctDCS combined with motor training could enhance motor gains in patients with stroke by regulating the functional connectivity of the target brain network. The estimated beta connectivity has been reported to predict the response to tDCS and motor learning in healthy adults^16,20^, which is consistent with our findings. However, we found no significant improvement in motor gains and change in connectivity after btDCS. This is inconsistent with the findings of an functional MRI–based network analysis that btDCS could enhance motor skill learning and induce an increase in functional connectivity in the ipsilesional M1 and premotor cortex in patients with chronic hemiparetic stroke^31^. The reasons for this are uncertain but may be multifactorial, including the heterogeneity of stroke patients and differences in motor tasks.

This study has some limitations. As scalp EEG has relatively poor spatial resolution compared with MRI, the anatomical correspondence between the 62 EEG electrodes and specific brain regions is imperfect. Additionally, this study only explored cortical connectivity using the ipsilesional and contralesional M1 as seed regions. As tDCS may induce changes in cortical connectivity across the whole brain network, it is important to study the effect of tDCS from the perspective of the whole brain network in the future.

In summary, this single-blind crossover study demonstrates that the cortical connectivity, measured in terms of beta coherence, of the ipsilesional and contralesional M1 was correlated with baseline stroke-related characteristics, including upper limb motor impairment and poststroke duration, and with motor gains following atDCS applied to the ipsilesional M1 or ctDCS applied to contralesional M1. These findings support the potential of EEG-based measures of cortical connectivity as bio-markers of motor gains following tDCS in patients with stroke.

## Methods

### Participants

This study included 28 patients who are first diagnosis of unilateral subcortical ischemic stroke based on magnetic resonance imaging (MRI) findings and can complete the Jebsen-Taylor Hand Function Test (JTT). We excluded patients who are: ① pre-stroke disability, ② surgical treatment for stroke, ③ history of seizures and the use of medications affecting central nervous system excitability, ④ wounds on the scalp, ⑤ cognitive deficits (Mini-mental State Examination score < 24); ⑥ depressive disorders (Patient Health Questionnaire-9 score > 5), and ⑦ inability to verbally communicate and provide informed consent. Participants were assessed for functional impairment using the Upper Extremity Fugl-Meyer Assessment (UEFM), Modified Barthel Index (MBI), and Motor Activity Log (MAL).

Participants were comprehensively explained about the experiment, including the possibility of minor adverse effects related to tDCS, such as transient itching, burning, and prickling of the scalp.

### Study design and procedure

The clinical trial registered in Clinical Medicine Research Institution of Tianjin Union Medical Centre, Affiliated to Nankai University, and approved by the ethics committee of Nankai University (No. NKUIRB2018016). All methods were performed in accordance with the relevant guidelines and regulations. All participants or their guardians signed a written informed consent form before beginning the experiment.

This was a participant-blind, randomized, crossover study. Participants were randomly assigned to receive one of the following four tDCS montages over the M1: ① anodal transcranial direct current stimulation (atDCS) with the anode over the lesioned M1 and cathode over the lateral supraorbital region as reference, ② cathodal transcranial direct current stimulation (ctDCS) with cathode over the unaffected M1 and anode over the lateral supraorbital region as reference, ③ bilateral transcranial direct current stimulation (btDCS) with the anode over the lesioned M1 and cathode over the unaffected M1, and ④ sham tDCS with the anode over the lesioned M1 and cathode over the lateral supraorbital region as reference but no continuous current application. The location of M1 was set as C3 (left hemisphere) or C4 (right hemisphere) according to the international 10–20 EEG electrode placement system. Four sessions were provided with an interval of 7 days between sessions. The above four montages were set up as in our previous studies^39^.

**Figure 5:**
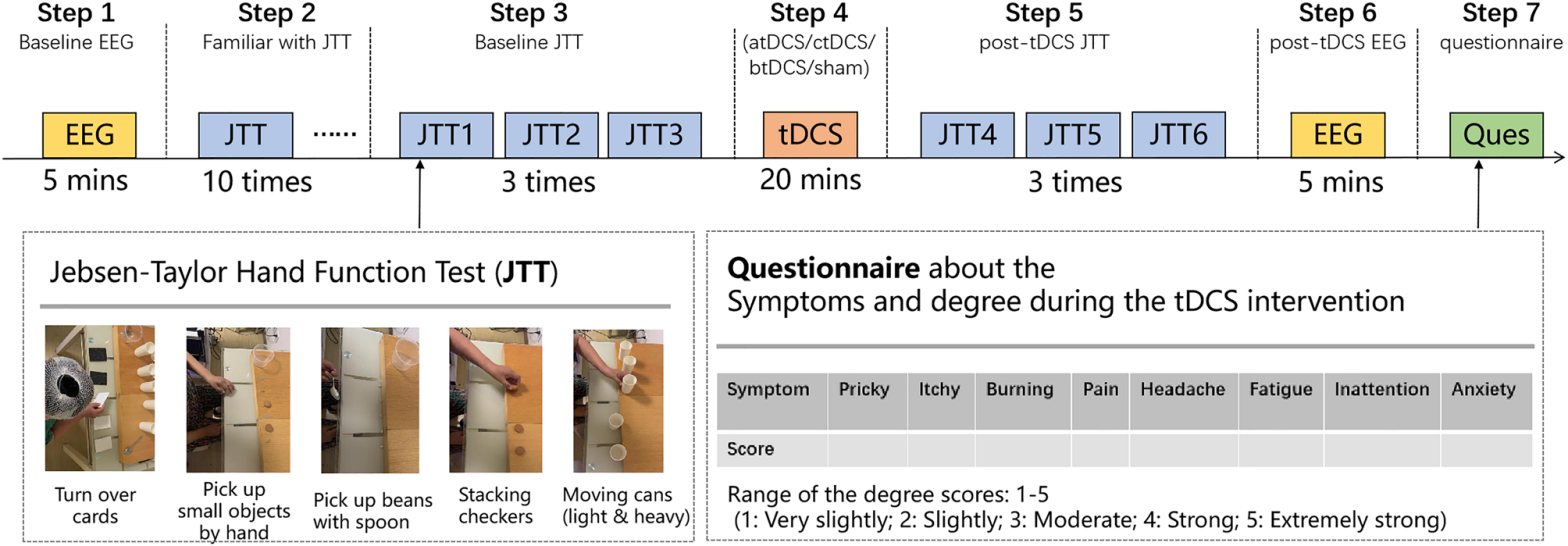
Experimental design. *EEG* electroencephalography, *JTT* Jebsen–Taylor Hand Function Test, *tDCS* transcranial direct current stimulation, *atDCS* anodal transcranial direct current stimulation, *ctDCS* cathodal transcranial direct current stimulation, *btDCS* bilateral transcranial direct current stimulation.

The Jebsen–Taylor Hand Function Test (JTT) was used to evaluate the function of the paretic hand. The four tDCS montage sessions were conducted as follows: Firstly, the baseline EEG signals were recorded (Step 1). Next, the participants were asked to practice the JTT 10 times to achieve a stable motor performance (Step 2). After the participants were familiar with the JTT, they performed it three times; the time taken to perform the test was noted, and the mean time of the three test performances was considered the baseline hand function (Step 3). tDCS was then applied, the montage of which was set based on the randomization results (Step 4). Subsequently, the JTT was performed three times, and the mean time was considered the post-tDCS hand function (Step 5). Thereafter, the post-tDCS EEG signals were recorded (Step 6). Finally, a questionnaire was administered to determine the participants’ symptoms during the stimulation (Step 7). The experimental design has been shown in Fig. 5.

tDCS was delivered using a battery-driven constant direct current (DC) stimulator (neuroConn, Germany) using a pair of 5 × 7-cm electrodes inserted into saline-soaked sponges. Stimulation was delivered at 1.75 mA (current density: 0.5 A/m^2^) for 20 min. In the sham tDCS, current was delivered only for the first 43 s (8 s ramp up, 30 s of DC stimulation, and 8 s ramp down) to make the participants feel a tingling sensation at the beginning of the stimulation.

### EEG acquisition and preprocessing

Resting-state EEG was performed with the eyes open for 5 min in a quiet and electromagnetically shielded room using the SynAmps2 EEG system (Neuroscan, US) with 62 Ag–AgCl electrodes placed over the scalp according to the international 10–20 system. Vertical and horizontal electrooculograms were recorded to enable the removal of ocular artifacts in a later processing step. The electrode impedance was maintained below 10 kΩ. The EEG signal was amplified with a band pass of 0.1–70 Hz and sampled at 1000 Hz. The forehead electrode was set as the ground electrode, and linked earlobes were set as reference electrodes. During EEG recording, the participants were asked to sit quietly in a comfortable chair, keep their eyes open, and fix their gaze at a point in front of them. They were also asked to relax and not engage in any cognitive or mental tasks during the EEG recording.

EEG data were exported to EEGLAB (a software toolbox of MATLAB) for pre-processing, which was performed as follows: Firstly, data were resampled to 250 Hz and filtered at 0.25–45 Hz using a third-order finite impulse response filter with the Hamming window method. Next, we visually selected a 100-s epoch that showed good quality. Finally, independent component analysis was performed to remove nonphysiological artifactual components, including eye blinks, cardiac activity, and scalp muscle activity.

The acquisition and pre-processing procedure were the same as in our previous study^39^.

### Coherence analysis

Coherence analysis was performed to evaluate the functional connectivity between brain regions and to provide additional information about the topography of synchronous oscillatory activity. The following formula was used, where x and y represent EEG signals from two different electrodes; *P*_*xx*_*(f)* and *Pyy(f)* are the power spectral densities of x and y; and *Pxy(f)* is their cross-spectral density:

$$C_{{{\text{xy}}}} (f) = \left| {P_{xy} (f)} \right|^{2} /P_{xx} (f)P_{yy} (f)$$

Coherence ranges from 0 to 1, with higher values indicating that the EEG signals have similar phase and amplitude. An increase in coherence may result from increased input from a tertiary common neural source^40^.

**Figure 6:**
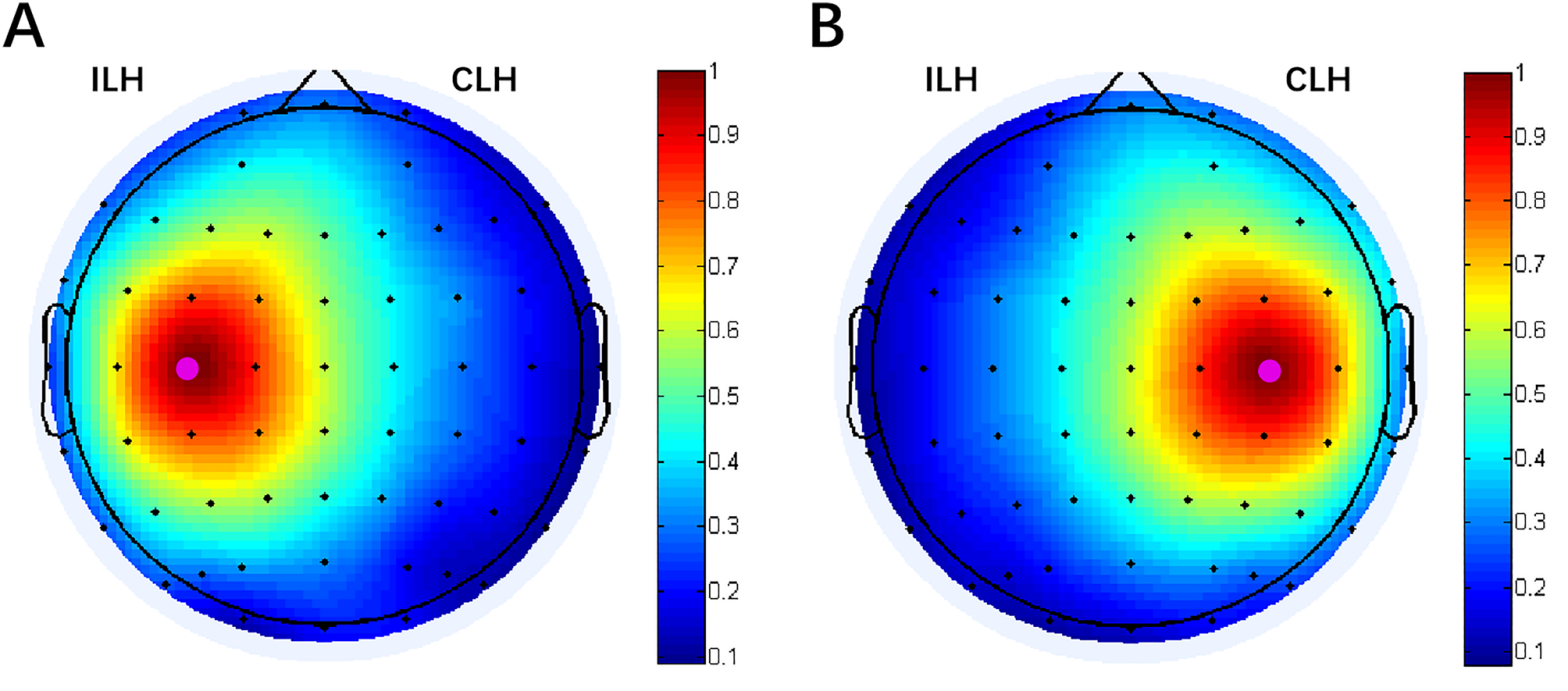
Topographic plot of beta coherence with the ipsilesional (**A**) and contralesional (**B**) M1 seeds. *ILH* ipsilesional hemisphere, *CLH* contralesional hemisphere.

The primary metric in this study was the coherence in beta frequency band (13–30 Hz). The coherence values were calculated between the seed regions of the ipsilesional or contralesional M1 and all other regions. Among the 62 electrodes of the international 10–20 electrode position system, the M1 seed was defined as either C3 (left hemispheric M1) or C4 (right hemispheric M1). In individuals with right hemisphere infarcts, coherence matrices were flipped across the midline for subsequent analyses^17^. Figure 6 shows the topographic plot of coherence with the ipsilesional and contralesional M1 seed (baseline).

### Statistical analyses

Statistical analyses were performed using MATLAB (MathWorks, Inc., Natick, MA), with statistical significance set as *p* < 0.05. All the data were normally distributed. Two-way repeated ANOVA was used to test the JTT performance after the four tDCS montages. The within-group effect, between-group effect and the interaction effect were analyzed by taking JTT Treatment as the within-group variable and Montage as the between-group variable. Mauchly sphericity test was performed before the above statistical analysis. If the sphericity assumption was not met, the statistical results were corrected using Greenhouse–Geisser method. If there was an interaction effect between the Treatment and the Montage, a separate effect analysis was performed. The average beta coherence from the pre-stimulation EEG was considered the baseline connectivity. Pearson correlation analysis was performed to investigate the relationship between clinical characteristics (age, UEFM score, poststroke duration, and MBI score) and baseline connectivity. The Benjamini–Hochberg method was used to decrease the false discovery rates and correct for multiple comparisons. Correlation analyses were performed to determine the association of upper limb motor gains with the change of cortical connectivity induced by atDCS, btDCS, ctDCS, and sham tDCS, with corrections for multiple comparisons performed using the Benjamini–Hochberg method. We removed the outliers before conducting the above analysis. As our data follows a normal distribution, outliers are defined as out of the range of mean ± 2.5 standard deviations. As poststroke duration affects functional connectivity according to the baseline analyses, we performed partial correlation analyses after controlling for poststroke duration when conducting the above correlation analyses.

## Ethics approval and consent to participate

This study was approved by the ethics committee of Nankai University (No. NKUIRB2018016) and have been performed in accordance with the Declaration of Helsinki. All methods were performed in accordance with the relevant guidelines and regulations. All participants or their guardians signed a written informed consent form before beginning the experiment.

### Supplementary Information

Supplementary Information.

## Data Availability

The EEG data and clinical characteristics of stroke patients are restricted by the Tianjin Union Medical Centre, in order to protect participants’ privacy. Data is available from corresponding author of the paper for researchers who meet the criteria for access to the confidential data.
