# Supplementary material for: Transcranial direct current stimulation–induced changes in motor cortical connectivity are associated with motor gains following ischemic stroke
Source: Sci Rep. 2024 Jul 8;14:15645. doi: 10.1038/s41598-024-66464-5 (PMC11231232; doi:10.1038/s41598-024-66464-5)
Supplement: Supplementary file 1 — Supplementary Information. [file 41598_2024_66464_MOESM1_ESM.docx]

Supplementary Table 1 Tests of Within-Subjects Effects

| Sources | Type Ⅲ Sum of Squares | df | F | Sig |
| --- | --- | --- | --- | --- |
| Montage | 431.067 | 3 | 0.291 | .832 |
| Time | 256.648 | 2 | 0.188 | .829 |
| Montage*Time | 852.160 | 6 | 0.243 | .961 |


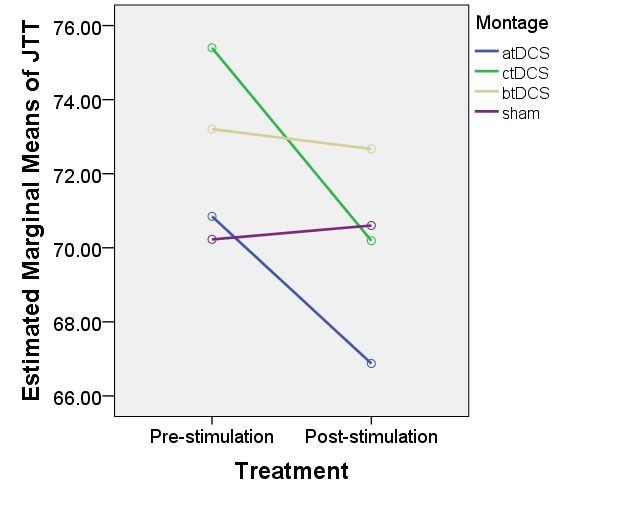


Supplementary Figure 1 Estimated marginal means of JTT time of the four tDCS montages


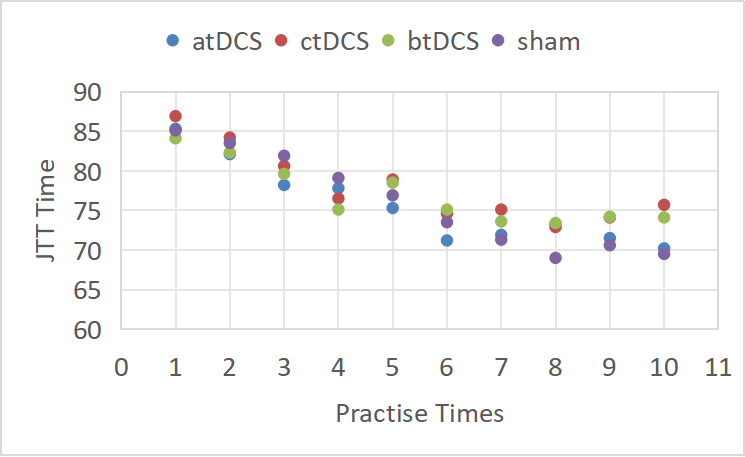


Supplementary Figure 2 Ten times practise result of JTT time of the four tDCS montages
